# Impact of cannabis decriminalization on selected psychotic disorders: a retrospective analysis at the Center for Mental Health and Prevention of Addiction (2013–2023) in Tbilisi

**DOI:** 10.1192/j.eurpsy.2026.10495

**Published:** 2026-07-31

**Authors:** L. Jishkariani, D. Zurabashvili, K. Gigolashvili, N. Jibuti, K. Jmukhadze, G. Lezhava

**Affiliations:** 1 Psychiatry, Tbilisi State Medical University; 2 Psychiatry, Tbilisi State University, Tbilisi, Georgia

## Abstract

**Introduction:**

Cannabis decriminalization raises concerns about its psychiatric impact, particularly on psychosis risk. As cannabis became decriminalized in Georgia in 2018, questions emerged regarding changes in substance-related and acute psychotic disorders. Examining these trends is essential to guide prevention, treatment, and policy in a shifting drug landscape.

**Objectives:**

This study aims to:

1. assess changes in rates of substance-associated psychoses following Georgia’s 2018 cannabis decriminalization;
2. examine diagnostic trends in acute/transient psychoses and cannabinoid-related disorders;
3. explore clinical and policy implications of observed patterns.

**Methods:**

A retrospective review was conducted of all adult psychiatric inpatient admissions (n = 12,680) at the Tbilisi Mental Health and Prevention of Addiction Center between 2013 and 2023. Unique first-time cases diagnosed with F23.0 (n = 789), F20.0 (n = 2,359), and F06.2 (n = 490) were extracted. Cases were stratified into pre-decriminalization (2013–2018) and post-decriminalization (2019–2023) periods. Substance use status, sex distribution, and diagnostic trends were analyzed descriptively.

**Results:**

Following decriminalization, the number of unique ATPD cases tripled (from 195 to 594). Substance-related psychotic cases increased fourfold (from 243 to 907), with cannabis implicated in over 70% of post-2018 substance cases. The proportion of psychosis cases involving substance use rose from 9.6% pre-2018 to 81.4% post-2018. Cannabis-only use increased 4.2-fold; polysubstance use increased 3.2-fold. Substance-using cases were overwhelmingly male (96%). F20.0 and F06.2 incidence showed a relative decline, potentially reflecting chronicity and under-detection in inpatient settings.

**Image 1:**
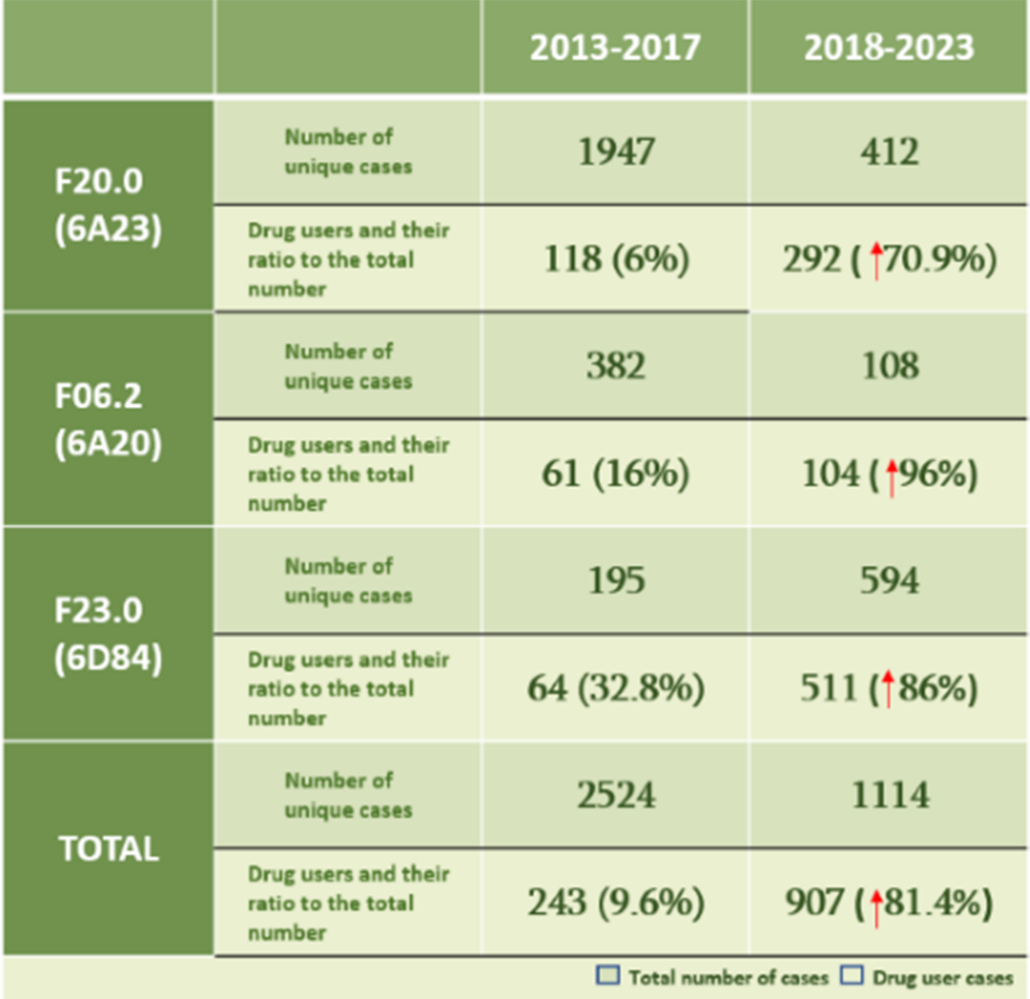
Image 1: Long description.

**Conclusions:**

A significant post-decriminalization rise in ATPD, predominantly among substance-using males, suggests a potential link between cannabis policy changes and psychosis incidence. The findings highlight the need for evidence-based prevention strategies, gender-responsive interventions, integrated dual-diagnosis care, and robust national monitoring systems.

**Disclosure of Interest:**

None Declared

## Long descriptions

### Image 1: Long description

The table consists of three main columns: Category, Metric, 2013-2017, and 2018-2023.

* Category F 2 0 dot 0 (6 A 2 3): Number of unique cases dropped from 1947 to 412. Drug users and their ratio increased from 118 (6%) to 292 (upward red arrow 70.9%).

* Category F 0 6 dot 2 (6 A 2 0): Number of unique cases dropped from 382 to 108. Drug users and their ratio increased from 61 (16%) to 104 (upward red arrow 96%).

* Category F 2 3 dot 0 (6 D 8 4): Number of unique cases increased from 195 to 594. Drug users and their ratio increased from 64 (32.8%) to 511 (upward red arrow 86%).

* TOTAL: Total unique cases decreased from 2524 to 1114. Total drug users and their ratio increased from 243 (9.6%) to 907 (upward red arrow 81.4%).

A legend at the bottom indicates dark blue squares represent Total number of cases and light blue squares represent Drug user cases.

Navigate back to Image 1:.

